# Normative Values for Spinopelvic Sagittal Parameters in the Asymptomatic Indian Population

**DOI:** 10.7759/cureus.91734

**Published:** 2025-09-06

**Authors:** Bharat R Dave, Abhijith Anil, Mirant B Dave, Sandesh Subhash Agrawal, Shivanand C Mayi, Ajay Krishnan, Ravi Ranjan Rai, Akruti Dave

**Affiliations:** 1 Spine Surgery, Stavya Spine Hospital and Research Institute, Ahmedabad, IND; 2 Orthopaedics/Spine Surgery, Pushpagiri Institute of Medical Sciences and Research Centre, Thiruvalla, IND; 3 Spine Surgery, Bhavnagar Institute of Medical Science, Bhavnagar, IND; 4 Spine, Stavya Spine Hospital and Research Institute, Ahmedabad, IND; 5 Physical Medicine and Rehabilitation, Stavya Spine Hospital and Research Institute, Ahmedabad, IND

**Keywords:** age more than 50, lumbar lordosis, normal value, pelvic incidence, pelvic tilt, sacral slope, sagital balance, thoracic kyphosis

## Abstract

Background

Accurate assessment of sagittal spinal and spinopelvic alignment is critical for evaluating adult spinal deformity (ASD) and optimizing surgical outcomes. However, most existing normative data and planning formulas are derived from Western populations, with limited data available from older Indian adults. This study aims to define normative values of sagittal alignment and spinopelvic parameters in asymptomatic Indian adults over 50 years of age and to develop population-specific regression models for preoperative planning in spinal deformity correction.

Methods

A prospective observational study was conducted on 250 asymptomatic Indian adults aged over 50 years (164 men, 86 women; mean age 59.95 ± 7.78 years). Standing whole-spine lateral radiographs were used to measure sagittal spinal and spinopelvic parameters. Pearson's correlation and regression analyses were performed to identify inter-parameter relationships and derive prediction equations.

Results

The mean values were: pelvic incidence (PI), 50.95° ± 11.72, pelvic tilt (PT), 13.0° ± 7.12, sacral slope (SS), 38.11° ± 9.71, lumbar lordosis (LL), 54.9° ± 13.7, thoracic kyphosis (TK), 29.96° ± 9.66, cervical lordosis (CL), -10.79° ± 11.99, and sagittal vertical axis (SVA), −0.26 mm ± 32.16. The following regression equations were derived: LL = 0.687 × PI + 19.9, PT = 0.326 × PI − 3.617, SS = 0.633 × PI + 5.854. A significant negative correlation was found between TK and LL (r = −0.268, p < 0.01), while a weak, non-significant correlation was observed between CL and LL. A PI-LL mismatch >9° was present in 44% of participants, and 10.8% had sagittal imbalance (SVA >50 mm). Cervical morphology was straight in 49.6%, lordotic in 26%, sigmoid in 19.6%, and kyphotic in 4.8% of subjects.

Conclusion

This study establishes normative values and population-specific planning formulas for sagittal and spinopelvic parameters in Indian adults aged 50 years and above. These equations offer more tailored targets for deformity correction than generalized Western models, particularly in individuals with extreme PI values. Adopting individualized alignment goals may enhance surgical planning and improve outcomes in ASD.

## Introduction

India has experienced a significant rise in life expectancy, increasing from approximately 37 years in 1950 to over 69 years by 2021 [1]. However, this rise in lifespan has not been accompanied by a proportionate increase in disease-free years. Studies have shown that nearly 16-20% of life is spent in morbidity during older age [2,3]. Among musculoskeletal causes of disability, back pain remains the leading contributor to years lived with disability globally [4]. Surgical correction of spinal deformities ranks among the most expensive procedures in orthopaedic and neurosurgical practice, with the average cost for primary deformity surgery exceeding USD 100,000 [5]. In such cases, the restoration of sagittal alignment plays a pivotal role in determining surgical outcomes and long-term satisfaction.

The burden of disability associated with adult spinal sagittal deformity (ASSD) increases with age and can lead to quality-of-life scores worse than those associated with chronic heart, pulmonary, or metabolic diseases [6]. Although normative values for spinopelvic alignment have been widely studied, most available data are derived from Western populations. While Indian studies have contributed to the literature, many are limited by small sample sizes [7,8], and the majority focus on younger individuals, thus reducing their relevance to the ageing population most affected by degenerative deformities.

Evaluation of spinopelvic parameters is essential not only for deformity correction but also for effective preoperative planning, functional restoration, and reduction of postoperative complications. Key parameters, such as pelvic incidence, pelvic tilt, sacral slope, lumbar lordosis, and sagittal vertical axis, are critical for assessing global alignment. A mismatch between pelvic incidence (PI) and lumbar lordosis (LL) (PI-LL mismatch) has been identified as a major predictor of mechanical complications and poor outcomes following deformity correction [9].

Over the past two decades, definitions of ideal spinal alignment have evolved, emphasizing individualized correction strategies based on a patient's pelvic anatomy. Schwab et al. demonstrated that achieving age-adjusted PI-LL targets significantly correlates with better health-related quality of life (HRQoL) [10]. However, most existing normative data and models are based on Western populations, which may not account for anatomical, ethnic, and lifestyle differences unique to Indian individuals. We stress the importance of population-specific studies like ours to enhance accuracy in preoperative planning and surgical outcomes.

Cultural and habitual postures common in India, such as squatting and cross-legged sitting, affect pelvic orientation and spinal curvature, particularly in the elderly. This underscores the importance of establishing normative reference values specific to this demographic. Although there are Indian studies that offer valuable insights, their focus on younger cohorts limits applicability to older adults [7-13]. This study aims to address that gap by establishing normative values for spinopelvic parameters in asymptomatic, community-dwelling Indian adults aged ≥50 years, thus supporting more accurate spinal deformity evaluation and surgical planning in the Indian context.

## Materials and methods

This prospective observational study was conducted at Stavya Spine Hospital & Research Institute, Ahmedabad, India, from June 2023 to June 2024. The study protocol was approved by the Institutional Ethics Committee of Stavya Spine Hospital & Research Institute (Protocol No.: SSHRI/CS/NS/SagPAR/BRD/61.1/6.23).

Participants

Asymptomatic volunteers aged over 50 years were recruited following a screening physical examination by an orthopaedic surgeon to rule out any pathology involving the spine, hip, knee, or ankle, until a total of 250 participants were enrolled. Individuals with a history of chronic back pain, spinal disorders, prior spine or lower limb surgeries, or limb length inequality were excluded.

Data collection

Radiographs were obtained with participants in a relaxed, free-standing posture without support, with hands positioned at the clavicle (elbows fully flexed, hands in a relaxed fist, wrists flexed, and the proximal interphalangeal joints resting in the supraclavicular fossae). This posture minimizes compensatory mechanisms that may obscure deformity and ensures visualization of all landmarks required to assess spinopelvic parameters. A standardized 36-inch cassette was used in all participants, with the X-ray source placed 2.5 metres from the film. Radiographs were centred at the T12 vertebra and captured during maximal inhalation, ensuring inclusion of the entire spine and pelvis from the occiput to the femoral heads. All radiographs were loaded onto Surgimap software v2.3.2.1 (Nemaris, Inc., New York, United States) for analysis.

Spinopelvic Parameters

The following spinopelvic parameters were independently measured by two authors (AA and BRD), and the mean of their observations was recorded: (i) Sagittal vertical axis (SVA): Distance between a vertical line from the center of C7 and the posterosuperior corner of S1; (ii) PI: Angle between the perpendicular to the S1 upper endplate and a line joining its center to the femoral head axis; (iii) Pelvic tilt (PT): Angle between the vertical and the line from the femoral head center to the midpoint of the S1 upper endplate; (iv) Sacral slope (SS): Angle between the horizontal and a line tangential to the S1 upper endplate; (v) LL: Cobb angle between the perpendiculars to the upper endplates of L1 and S1; (vi) Thoracic kyphosis (TK): Angle between the perpendiculars to the T4 upper and T12 lower endplates; (vii) Cervical lordosis (CL): Angle between the perpendiculars to the lower endplates of C2 and C7; (viii) Thoracolumbar angle (TLA): Angle between the upper endplate of T10 and the lower endplate of L2; (ix) Cervical SVA: Distance between a vertical line dropped from the centroid of C2 and the posterosuperior corner of T1; (x) Neck tilt (NT): Angle between the vertical and a line from the upper end of the sternum to the midpoint of the T1 upper endplate; (xi) Thoracic inlet angle (TIA): Angle between the line from the sternum to the centre of the T1 upper endplate and a line perpendicular to the T1 upper endplate; (xii) T1 slope (T1S): Angle between a horizontal line and the superior endplate of T1. If T1 was inadequately visualized, the C7 upper endplate was used instead.

Cervical Spine Morphology

Cervical spine morphology was assessed on lateral radiographs by drawing a reference line between the midpoints of the C2 and C7 endplates. The classification of cervical spine morphology was based on the method described by Le Huec et al. [14], which categorizes the cervical spine into lordotic, straight, kyphotic, or sigmoid based on the position of centroid points. The spine was classified as: 

Lordotic: When the centroids of C3-C6 vertebrae lie anterior to the reference line with an apex >2 mm anterior

Straight: When centroids lie close to the line and the apex is within 2 mm

Kyphotic: When at least one centroid is >2 mm posterior to the line

Sigmoid: When centroids were variably located anterior and posterior to the line, with at least one centroid is >2 mm from the reference

Data analysis

The sagittal alignment parameters of these asymptomatic volunteers were analyzed to derive normative values for individuals aged >50 years. Data normality was evaluated using the Kolmogorov-Smirnov test due to the large sample size. In addition to the regression analyses for PI-related parameters (LL, PT, SS), Pearson's correlation analysis was conducted to examine the relationships between CL, TK, and LL. The significance of the correlations was determined using p-values, with values less than 0.05 considered statistically significant. For variables that followed a normal distribution, simple linear regression was employed to assess associations. A p-value below 0.05 was regarded as indicative of statistical significance. All data analyses were performed using IBM SPSS Statistics for Windows, version 20.0 (Released 2011; IBM Corp., Armonk, New York, United States). The strength of correlations was classified as strong (>0.5), moderate (>0.3), or small (>0.1).

## Results

A total of 250 asymptomatic Indian adults over 50 years of age were included in the study, comprising 164 men and 86 women. The mean age was 59.95 ± 7.78 years. The mean sagittal balance parameters are summarised in Table 1.

**Table 1:** Parameters of sagittal balance in study population cSVA: cervical sagittal vertical axis

Parameters	Mean	Std. Deviation
Sagittal vertical axis (mm)	-0.26	32.16
Cervical lordosis (degrees)	-10.79	11.99
Thoracic kyphosis (degrees)	29.96	9.66
Thoracolumbar angle (degrees)	10.79	10.27
Lumbar lordosis (degrees)	-54.90	13.72
Sacral slope (degrees)	38.11	9.71
Pelvic tilt (degrees)	13.00	7.12
Pelvic incidence (degrees)	50.95	11.72
T1 slope (degrees)	27.76	9.13
Neck tilt (degrees)	59.76	7.44
Thoracic inlet angle (degrees)	87.51	10.34
cSVA (mm)	25.78	12.31
Chin brow vertical angle (degrees)	2.11	8.01

Regression analysis was performed to evaluate the relationship between pelvic incidence (PI) and other spinopelvic parameters. The following statistically significant equations (p < 0.001) were derived:

LL = [0.687 x PI] + 19.9

PT = [0.326 x PI] - 3.617

SS = [0.633 x PI] + 5.854

Pearson’s correlation analysis revealed a significant negative correlation between TK and LL (r = −0.268, p < 0.01). In contrast, the correlation between CL and LL was statistically significant but weak (r = -0.268, p < 0.01). However, a statistically significant weak negative correlation was observed between the values of thoracic kyphosis and lumbar lordosis.

Cervical spine morphology

On lateral X-ray assessment, the cervical spine morphology among the study participants was categorized into four distinct shapes. CL was observed in 26% of subjects (65 out of 250), while nearly half of the participants (49.6%, 124 out of 250) exhibited a straight cervical spine. A smaller proportion, 4.8% (12 out of 250), demonstrated a kyphotic cervical spine, and 19.6% (49 out of 250) had a sigmoid-shaped cervical spine.

Prevalence of sagittal imbalance and PI-LL mismatch

Among the participants, 27 subjects were found to have a sagittally unbalanced spine, defined as SVA > 50 mm from the postero-superior corner of the S1 endplate. Of these, six had negative sagittal balance, and 21 had positive sagittal balance. Pelvic imbalance, defined as PT > SS, was noted in four of the 250 subjects. A PI-LL mismatch > 9° was observed in 110 (44%) subjects.

## Discussion

Studies correlating sagittal balance of the spine to quality of life have primarily been performed in Western countries [5,6,9,10]. There is limited literature on the subject in India, and studies assessing the normative values of sagittal balance parameters in an Indian population are hindered by small sample sizes. The majority of the literature on the subject focuses on sagittal balance parameters in the young [11-13]. In contrast, the majority of patients with adult spinal deformity and symptomatic lumbar pathology belong to the older age group [1-4,6,9,10].

Our study analyzed sagittal balance and spinopelvic parameters in 250 asymptomatic subjects aged 50 years or older. The mean (± SD) sagittal vertical axis in our subjects was -0.26 (32.16) mm, which was comparable to the values reported in most studies conducted both abroad and in India [7-10,14]. The only other study that reported the mean sagittal vertical axis in an Indian population studied 100 asymptomatic volunteers and found a mean value of 15.6 mm (± 6.9 mm) [8]. Our study, despite involving only volunteers over the age of 50 years, did not observe an increase in the SVA value, which would have indicated an increase in kyphosis. The mean cervical lordosis was 10.79 degrees (± 11.99 degrees), Thoracic kyphosis was 29.96 degrees (± 9.66 degrees), and lumbar lordosis was 54.9 degrees (± 13.7). These values align with the reported values in the literature for both Western and Indian populations, as shown in Table 2 and Table 3, respectively. 

**Table 2:** Normative values of spinopelvic parameters (studies outside India)

Author, year	Pelvic incidence(degrees)	Sacral slope (degrees)	Pelvic tilt (degrees)	Lumbar lordosis(degrees)	Thoracic kyphosis (degrees)	SVA (cm)
Pratali et al., 2014 [15]	48.7 ± 9.6	38 ± 8.4	12.15 ± 6.2	-	-	-1.74 ± 3.7
Hammerberg and Wood, 2003 [16]	60.5 ± 15.2	42 ± 9.6	17.7 ± 9.1	57.4 ± 13.7	52.5 ± 12.2	4.04 ± 3.73
Hasegawa et al., 2016 [17]	52.3 ± 11.1	40.8 ± 8.5	11.5 ± 7.6	55.4 ± 11.2	41.5 ± 9.9	0.1 ± 2.3
Price et al., 2016 [18]	50.1 ± 11	38.8 ± 8	11.5 ± 6.3	56.8 ± 9.7	-	-
Yukawa et al., 2016 [19]	53.7 ± 10.9	39.4 ± 8	14.5 ± 8.4	49.7 ± 11.2	36 ± 10.1	3.1 ± 12.6
Schwab et al., 2006 [20]	52 ± 10	30 ± 9	15 ± 7	60 ± 12	41 ± 12	- 20 ± 30
Berthonnaud et al., 2005 [21]	51 ± 5.3	49.7 ± 4.1	12.1 ± 3.2	42.7 ± 5.4	47.5 ± 4.8	
Vialle et al., 2005 [22]	54.7 ± 10.6	41.2 ± 8.4	13.2 ± 6.1	60.2 ± 10.3	40.6 ± 10	-
Legaye et al., 1998 [23]	52 ± 10	40 ± 8.5	11 ± 5.5	60 ± 10	43 ± 13	-
Boulay et al., 2006 [24]	53.1 ± 9	41.2 ± 7	12 ± 6.4	66.4 ± 9.5	53.8 ± 10.1	-
Roussouly et al., 2006 [25]	50.6 ± 10.2	39.6 ± 7.6	11.1 ± 5.9	61.2 ± 9.4	46.3 ± 9.5	-
Rezaee et al., 2020 [26]	51.5 ± 10.9	34.8 ± 8.8	17.4 ± 9.9	44.6 ± 9.8	34 ± 12.3	-
Mac-Thiong et al., 2011 [27]	52.6 ± 10.4	39.6 ± 7.9	13 ± 6.8	-	-	-
Lee et al., 2015 [28]	45.6 ± 9.3	33.4 ± 8	-	47.3 ± 9.8	28.5 ± 9	-
Nunez-Pereira et al, 2015 [29]	54.3 ± 13.6	30.8 ± 14.3	24 ± 10.9	46.2 ± 15.2	34.3 ± 17.1	30.4 ± 39.9*
Tang et al., 2021 [30]	47.8 ± 8.3	37.4 ± 7	10.3 ±6.2	48 ± 9.7	24.6 ± 7.1	-21.6 ± 31

**Table 3:** Normative values of spinopelvic parameters (studies from India)

Author, year (sample size)	Mean age (years)	Pelvic incidence (degrees)	Sacral slope (degrees)	Pelvic tilt (degrees)	Lumbar lordosis (degrees)	Thoracic kyphosis (degrees)	SVA(cm)
Bhosale et al., 2019 [7] (n = 130)	34.49 ± 8.53	51.5 ± 6.85	39.2 ± 6.3	12.32 ± 5.4	-	-	-
Sudhir et al., 2016 [8] (n= 101)	47.16 for men, 48.59 for women	55.4 ± 5.31	35.99 ± 7.53	17.97 ± 7.16	48.84 ± 9.82	32.55 ± 10.92	-
Ganesan et al., 2014 [9] (n=120)	30	58.64 ± 12.59	41.2 ± 11.01	14.2 ± 7.32	-	-	-
Siddiqui et al., 2015 [12] (n=84)	Not reported	49.4 ± 7.6	37.4 ± 6.6	13.9 ± 5.8	-	-	-
Singh et al., 2016 [11] (n=50)	31.14 ± 9.62	48.52 ± 8.99	39.14 ± 7.05	9.3 ± 7.16	58.78 ± 9.51	-	-
Sangondimath et al., 2022 [10] (n=100)	27.52 ± 6.13	51.28 ± 11.63	39.62 ± 7.98	12.46 ± 7.75	43.61 ± 12.04	35.04 ± 8.74	1.56 ± 6.49
Current Study, 2025 (n = 250)	59.95 ± 7.78	50.95 ± 11.72	38.11 ± 9.71	13 ± 7.12	54.9 ± 13.72	29.96 ± 9.66	-0.26 ± 3.2

With regard to the pelvic parameters, the average PI, SS, and PT were 50.95 degrees (± 11.72), 38.11 degrees (± 9.71), and 13 degrees (± 7.12), respectively. These values also match the reported values for these parameters, which range from 48.5 to 58.6 degrees for PI, 37.4 to 41.2 degrees for SS, and 9.3 to 17.97 degrees for PT in the Indian population. The ranges of normative values for these parameters reported in the Western literature are similar. Other studies comparing Caucasian and Japanese populations have found the values of PI, PT, and SS to be comparable [17]. At the same time, differences were noted in LL and TK. This difference was attributed to the tendency of Asian populations to squat more, which could have improved the hip extension reserve in Asian subjects [30].

Regression analysis found the following relationships between these parameters:

LL = [0.687 x PI] + 19.9

PT = [0.326 x PI] - 3.617

SS = [0.633 x PI] + 5.854

**Figure 1:**
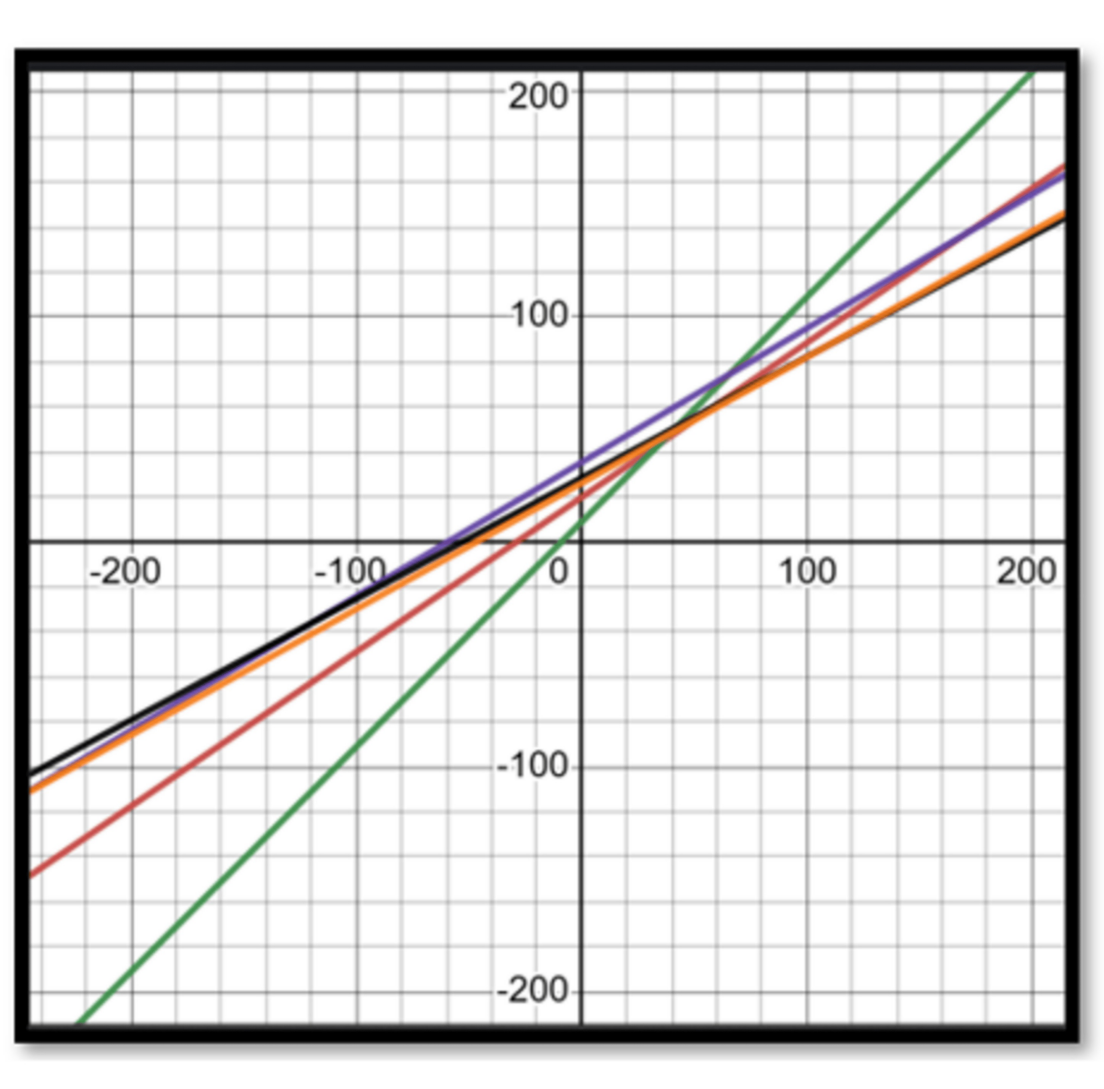
Regression equations – lumbar lordosis and pelvic incidence Red: current study; Green: Schwab et al. [20]; Blue: Legaye et al. [23]; Orange: Hasegawa et al. [17]; Black: Le Huec and Hasegawa [31]

We compared the relationships derived from the regression analysis of our subjects to those found by regression analysis using models of Hasegawa et al. [17], Price et al. [18]**, **Schwab et al. [20], Legaye et al. ​[23]**, **andLe Huec and Hasegawa [31]**.** The graphs of the different regression equations highlighting the relation between LL and PI are shown in Figure 1.

**Figure 2:**
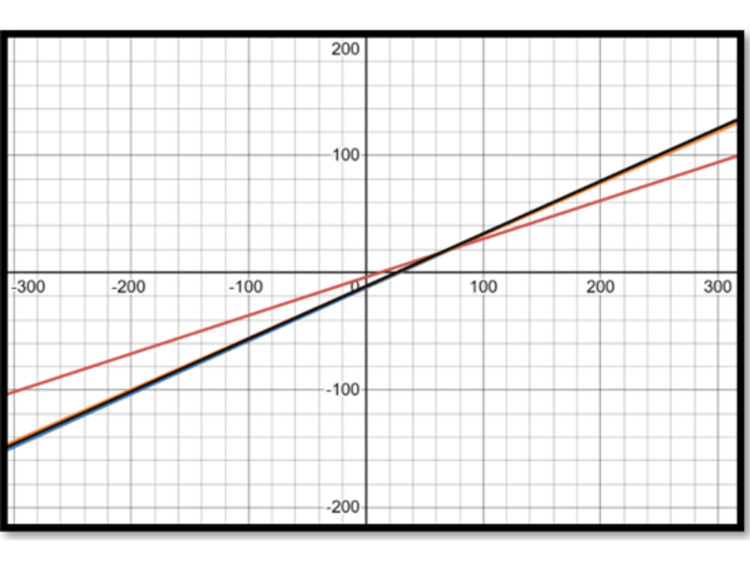
Regression equations – pelvic tilt and pelvic incidence Red: current study; Blue: Legaye et al. [23]; Orange: Hasegawa et al. [17]; Black: Le Huec and Hasegawa [31]

**Figure 3:**
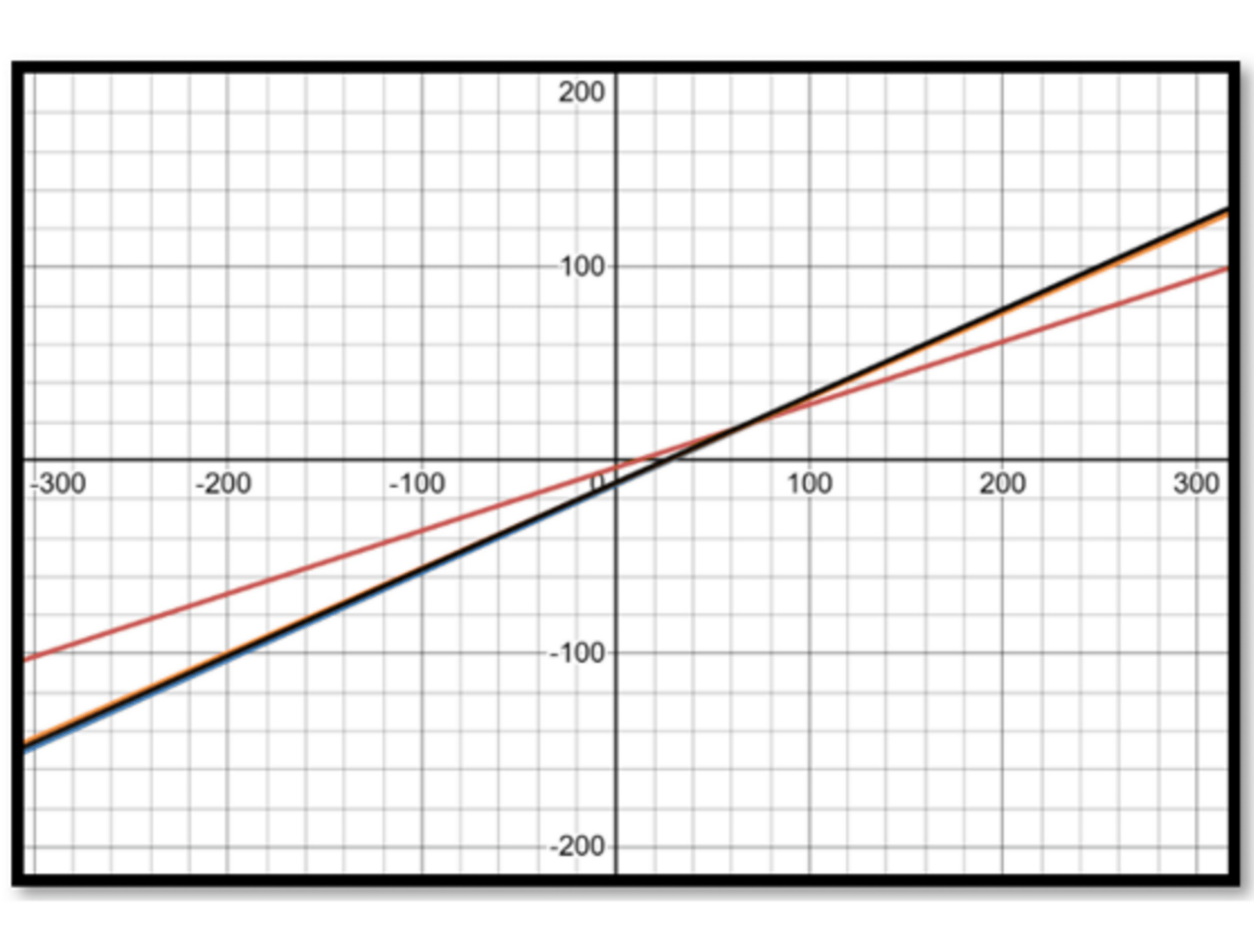
Regression equations – sacral slope and pelvic incidence Red: current study; Blue: Legaye et al. [23]; Orange: Hasegawa et al. [17]; Black: Le Huec and Hasegawa [31]

Using population-specific equations to determine the ideal LL is likely to yield optimum results and minimize the chances of overestimating or underestimating the lordosis to be restored. Thus, an equation specific to the Indian population in patients over 50 years of age is essential for effective preoperative planning. Figures 2, 3 show graphs of the regression equations showing the relation of PI with PT and SS, respectively, given by different authors. This again highlights the importance of population-specific equations for preoperative planning, as the relationship between PI and the parameters of LL, PT, and SS is crucial. This difference gets accentuated, especially at higher values of PI. The use of individualized values for PT, tailored to the patient's PI, is essential. Using a single average value for PT (25 degrees, as given by Schwab et al. [20]) can yield suboptimal results, especially in patients with a small PI.

The normative values of each parameter observed among our subjects are also comparable to those observed by Schwab et al. [32] in their analysis of sagittal balance parameters in the age groups of 41-60 and in patients over 60 years old [20]. The average cervical SVA in our subjects was 25.79 mm (± 12.31), which is comparable to the values reported in the literature. Our subjects' mean CL is also comparable to the values reported in the literature by multiple authors, as summarised in Table 4.

**Table 4:** Cervical sagittal balance parameters

Study	Mean Age (years)	C0 – C7 lordosis (degrees)	C2 – C7 lordosis (degrees)	C2 – C7 SVA (mm)	T1 slope (degrees)	C2 tilt (degrees)	Thoracic inlet angle (degrees)	Neck tilt (degrees)
Tang et al. [33] (n=126)	23.2 ± 4.4	26 ± 12.8	10.6 ± 13.6	18.6 ± 7.9	26.1 ± 7	-	-	-
Le Huec and Hasegawa [31] (n= 106)	38.13	-	4.89 ± 12.84	-	19.64 ± 8.76*	10.48 ± 6.93	-	-
Lee et al. [28] (n= 77)	31.5 ± 7.6	-	9.9 ± 12.5	-	25.7 ± 6.4	-	69.5 ± 8.6	43.7 ± 6.1
Nunez-Pereira et al. [29] (n=145, only 34 asymptomatic)	53.6 ± 13.4	-	15.8 ± 13.2	-	23.4 ± 11.7*	-	-	-
Theologis et al. [34] (n= 87)	49 ± 16	-	11 ± 14	21 ± 9	25 ± 9	-	-	-
Iyer et al. [35] (n= 115)	50.1	-	12 ± 14	21 ± 12	26 ± 9	-	-	-
Current Study (n = 250)	59.95 ± 7.78	-	10.79 ± 11.99	25.78 ± 12.31	27.76 ± 9.13	-	87.51 ± 10.34	59.76 ± 7.44

The normative values observed in our study are comparable to those reported in other countries [35], suggesting some degree of variability but overall alignment with existing literature. The T1 slope reported in other studies from Western countries and East Asia reports values between 17 and 25 degrees (Table 4). The average neck tilt in patients in the current study was approximately 60 degrees, which differs significantly from the 44 degrees postulated by Lee et al. [28] as the optimum value of neck tilt to minimize energy expenditure. This could signify an ethnic difference between the two study populations, and further research is needed to examine this critical difference.

A statistically significant negative correlation (Pearson's coefficient -0.268) was noted between TK and LL. While the negative correlation is logical, with the mobile lumbar spine compensating for any increased TK to maintain global spine alignment, the small coefficient signifies that LL is affected not just by TK but is influenced by other parameters as well. Interestingly, 27 of the 250 asymptomatic patients had an unbalanced spine, four patients had an unbalanced pelvis, and 110 of the 250 patients were noted to have a mismatch between the PI and LL greater than 9 degrees. These figures indicate that Schwab's criteria for determining the mismatch between pelvic incidence and lumbar lordosis need to be revisited.

When analyzing the shapes of the cervical spine, we noted that the majority of our patients had a straight alignment. In a cohort of 120 asymptomatic adults with a mean age of 23 years, Ganesan et al. found that 45.8% had straight cervical spines, 28.3% had lordotic cervical spines, 21.7% had a kyphotic spine, and 4.2% had a sigmoid shape of the cervical spine [9]. Hardacker et al. studied the alignment of the cervical spine in 100 asymptomatic adult volunteers with a mean age of 38 years, and while they did not classify the shape of the cervical spine, they noted that cervical segmental kyphosis was typically present in 36% of patients [36]. The results of the current study suggest that with increasing age, a similar pattern of sagittal alignment is maintained. Boyle et al., in their cadaveric study, noted that CL tends to flatten with age, and the apex of lordosis tends to move cranially [37]. They also noted that the inflexion points between CL and TK migrated cranially with age.

Limitations and future directions

Our study is limited by the fact that all subjects were from a single centre, and our results may not apply to subjects from other parts of India. Other limitations include potential confounding factors such as BMI, habitual postures, physical activity, or undiagnosed subclinical conditions, which may have influenced the sagittal parameters. Future studies should consider these variables to better understand their impact on sagittal alignment. Further research into patient outcomes after deformity correction using these equations will validate the utility of the analyses done.

## Conclusions

This study establishes normative values for sagittal spinal and spinopelvic parameters in asymptomatic Indian adults over 50 years of age, addressing a gap in the literature that is largely based on Western populations. Deriving population-specific regression models, it offers tailored formulas for predicting LL, PT, and SS from pelvic incidence, thereby enhancing precision in preoperative planning and individualized surgical correction goals.

The findings highlight notable variations in sagittal alignment parameters within the Indian elderly population, underscoring the limitations of applying Western reference data to Indian patients. Adoption of these localized normative values and regression equations may improve surgical planning, optimize restoration of physiological sagittal balance, and potentially reduce complications and revision rates. These results also provide a benchmark for future research to explore correlations between spinopelvic alignment, functional outcomes, and quality of life in Indian patients undergoing deformity correction.
